# The Feasibility of Magnetic Resonance Imaging for Quantification of Liver, Pancreas, Spleen, Vertebral Bone Marrow, and Renal Cortex R2* and Proton Density Fat Fraction in Transfusion-Related Iron Overload

**DOI:** 10.4274/tjh.2015.0142

**Published:** 2016-02-17

**Authors:** İlkay S. İdilman, Fatma Gümrük, Mithat Haliloğlu, Muşturay Karçaaltıncaba

**Affiliations:** 1 Hacettepe University Faculty of Medicine, Department of Radiology, Ankara, Turkey; 2 Hacettepe University Faculty of Medicine, Liver Imaging Team, Ankara, Turkey; 3 Hacettepe University Faculty of Medicine, Department of Pediatrics, Division of Pediatric Hematology, Ankara, Turkey

**Keywords:** iron overload, liver, Pancreas, R2*, Magnetic resonance imaging-proton density fat fraction

## Abstract

**Objective:**

We aimed to evaluate the feasibility of quantification of liver, pancreas, spleen, vertebral bone marrow, and renal cortex R2* and magnetic resonance imaging-proton density fat fraction (MRI-PDFF) and to evaluate the correlations among them in patients with transfusion-related iron overload.

**Materials and Methods:**

A total of 9 patients (5 boys, 4 girls) who were referred to our clinic with suspicion of hepatic iron overload were included in this study. All patients underwent T1-independent volumetric multi-echo gradient-echo imaging with T2* correction and spectral fat modeling. MRI examinations were performed on a 1.5 T MRI system.

**Results:**

All patients had hepatic iron overload. Severe hepatic iron overload was recorded in 5/9 patients (56%), and when we evaluated the PDFF maps of these patients, we observed an extensive patchy artifact in the liver in 4 of 5 patients (R2* greater than 671 Hz). When we performed MRI-PDFF measurements despite these artifacts, we observed artifactual high MRI-PDFF values. There was a close correlation between average pancreas R2* and average pancreas MRI-PDFF (p=0.003, r=0.860). There was a significant correlation between liver R2* and average pancreas R2* (p=0.021, r=0.747), liver R2* and renal cortex R2* (p=0.020, r=0.750), and average pancreas R2* and renal cortex R2* (p=0.003, r=0.858). There was a significant negative correlation between vertebral bone marrow R2* and age (p=0.018, r=-0.759).

**Conclusion:**

High iron content of the liver, especially with a T2* value shorter than the first echo time can spoil the efficacy of PDFF calculation. Fat deposition in the pancreas is accompanied by pancreatic iron overload. There is a significant correlation between hepatic siderosis and pancreatic siderosis. Renal cortical and pancreatic siderosis are correlated, too.

## Abstract (tr)

**Amaç:**

Bu çalışmada, transfüzyon ilişkili demir birikimi bulunan hastalarda karaciğer, pankreas, dalak, vertebral kemik iliği ve böbrek korteksi R2* ve manyetik rezonans görüntüleme-proton dansite yağ oranı (MRG-PDYO) ölçümünün uygulanabilirliği ve bunlar arasındaki ilişkiyi değerlendirmeyi amaçladık.

**Gereç ve Yöntemler:**

Çalışmaya, karaciğer demir birikimi şüphesi ile kliniğimize gönderilmiş olan toplam 9 hasta (5 erkek, 4 kız) dahil edildi. Tüm hastalara T2* düzeltmeli ve spektral yağ modellemeli T1 bağımsız volumetrik multi-eko gradient-eko görüntüleme yapıldı. MRG incelemeleri 1.5 T MRG sistemi ile yapıldı.

**Bulgular:**

Tüm hastalarda karaciğerde demir birikimi mevcuttu. Dokuz hastanın beşinde (%56) ağır demir birikimi saptandı ve bu hastaların PDYO haritaları incelendiğinde 4 hastada (R2* değeri 671 Hz’den fazla olanlarda) karaciğerde yaygın yamasal artefaktlar olduğu saptandı. Bu artefaktlara rağmen MRG-PDYO ölçümü yapıldığında normalden çok yüksek MRG-PDYO değerleri belirlendi. Ortalama pankreas R2* ve ortalama pankreas MRG-PDYO arasında (p=0,003, r=0,860), karaciğer R2* and ortalama pankreas R2* arasında (p=0,021, r=0,747), karaciğer R2* ve renal korteks R2* arasında (p=0,020, r=0,750) ve ortalama pankreas R2* ve renal korteks R2* arasında (p=0,003, r=0,858) anlamlı korelasyon saptandı. Vertebral kemik iliği R2* ve yaş arasında anlamlı negatif korelasyon saptandı (p=0,018, r=-0,759).

**Sonuç:**

Karaciğerdeki yüksek demir birikimi özellikle de T2* değeri ilk eko zamanından daha kısa olduğunda PDYO hesaplamasını bozabilmektedir. Pankreastaki yağ birikimi pankreatik demir birikimine eşlik edebilmektedir. Karaciğer ve pankreas siderozisi birbiri ile ilişki göstermektedir. Böbrek korteksi ve pankreas siderozisi arasında da ilişki mevcuttur.

## INTRODUCTION

Iron is an essential nutrient for all human cells [1,2]. Under normal circumstances, intake and excretion of iron is balanced within a daily range of 1-2 mg [3]. There is no effective way of excretion of iron from the body; therefore, if the total amount of income exceeds outcome, such as in cases of increased intestinal absorption, long-term transfusion therapies, or excess parenteral iron treatment, total body iron increases. Transfusion-related iron overload is one of the leading causes of iron overload. It primarily depends on repetitive transfusions that bring a burden of excess iron, especially after 40 to 50 transfusions that saturate the capacity of reticuloendothelial system [4]. After saturation, iron accumulates in parenchymal organs like the liver, pancreas, myocardium, and endocrine glands, which leads to tissue damage and fibrosis [3].

Iron is mainly stored in the liver and the iron content of the liver is an indirect marker of total body iron [5]. Hence, quantification of hepatic iron content is used for guiding and monitoring iron chelation therapies in transfusion-related iron overload [1]. Current methods for quantification of hepatic iron consist of liver biopsy and imaging. Percutaneous liver biopsy is the current reference method for quantification of hepatic iron content. However, it is an invasive procedure that can bring serious complications, and acquired small sample sizes in liver biopsy are insufficient to represent the whole organ. In addition, iron overload can be organ-specific and estimation of iron accumulation in different organs such as the pancreas, spleen, vertebral bone marrow, and kidneys requires noninvasive techniques.

Magnetic resonance imaging (MRI) methods including signal intensity ratio and relaxometry are promising for quantification of hepatic iron overload compared with liver biopsy [6,7,8]. However, coexistence of fat influences the measurement of hepatic iron content because of the spectral complexity of fat [9]. Vice versa, hepatic iron overload influences the measurement of hepatic fat content in chemical shift techniques due to its T2* shortening effect [10]. A recent technique, MR IDEAL-IQ (Iterative Decomposition of water and fat with Echo Asymmetry and Least square estimation), which was defined primarily for quantification of the fat fraction of tissue, quantifies R2* by correcting the fat-dependent confounding factors [11]. However, the former technique’s accuracy was found to be lower, especially in patients with T2* values below 1 ms [12]. Additionally, the feasibility of this technique was not evaluated in the estimation of pancreas, spleen, vertebral bone marrow, and renal cortex iron overload. In the present study, we aimed to evaluate the feasibility of MR IDEAL-IQ in the quantification of liver, pancreas, spleen, vertebral bone marrow, and renal cortex R2* and MRI-proton density fat fraction (MRI-PDFF) and to evaluate the correlations among them in patients with transfusion-related iron overload.

## MATERIALS AND METHODS

### Patients

This was a retrospective cross-sectional study. A total of 9 patients (5 boys, 4 girls) who were referred to our clinic with suspicion of hepatic iron overload and examined with MR IDEAL-IQ between August 2010 and November 2010 were included in the study. All of the patients had a history of repetitive transfusions, 8 of them with a diagnosis of beta thalassemia major and 1 of them with non-Hodgkin lymphoma (NHL). Body mass index (BMI) was calculated as weight in kilograms divided by height in meters squared.

### Magnetic Resonance Imaging Examination

MR images were acquired with a 1.5 T HDxt MRI system (GE Healthcare, Milwaukee, WI, USA). The subjects were examined in the supine position. An 8-channel phased array body coil was used for acquisition. A 3-plane gradient echo localizer sequence was performed at the beginning of the examination. The MRI protocol included the IDEAL-IQ sequence. This is a 3D volumetric imaging sequence used to create T2* and triglyceride fat fraction maps from a single breath-hold acquisition. The technique was used to estimate R2* (1/T2*) and PDFF (water-triglyceride fat separation) in the liver simultaneously in a single acquisition. Afterwards, a correction was applied to the resulting PDFF maps to correct for T2* effects. Six gradient echoes were applied to reconstruct water and triglyceride fat images, relative triglyceride fat fractions, and R2* maps. The IDEAL-IQ sequence uses a novel “complex field map” to incorporate the T2* and field inhomogeneity effects into the overall multi-echo acquisition signal model. It was shown by Yu et al. [11] that, by acquiring a 6-echo image and estimating a complex field map using an iterative least square estimation algorithm, it is possible to achieve fat-water separation and T2* estimation in a single breath-hold acquisition.

The parameters of this sequence were TR: 12.9 ms, FOV: 35-40 cm, matrix: 224x160, 125 kHz bandwidth, flip angle: 5 °, and slice thickness: 5 mm. A single 3D slab with 44 to 56 slices was acquired. We acquired data sets with 6 different echoes ranging from 1.6 ms to 9.8 ms. A 2D self-calibrated parallel imaging technique called auto calibrating reconstruction of Cartesian sampling was used with an acceleration factor of 2. The images were processed using the software provided by the manufacturer to create water, fat, in-phase, opposed-phase, R2*, and fat fraction maps.

### Image Analysis

By using a work station (AW 4.4, GE Healthcare), a radiologist placed an elliptic region of interest (ROI) of approximately 4 cm2 in Couinaud segments 5-6 on the PDFF maps and the R2* maps, avoiding blood vessels, bile ducts, and artifacts. An elliptic ROI of approximately 1 cm2 for the pancreatic head, body, and tail on the PDFF maps and the R2* maps was placed for pancreatic measurements and the arithmetic mean was calculated. The same procedure was performed for the spleen with a ROI of 2 cm2, for the L1 or L2 vertebral corpus with a ROI of 2 cm2, and for the renal cortex with a ROI of 1 cm2. The patients with a T2* value under 18 ms were included in the hepatic iron overload group [13]. The patients with a T2* value under 2 ms were included in the severe hepatic iron overload group. The patients with pancreatic R2* values between 30 and 100 Hz were included in the mild pancreatic siderosis group, those between 100 and 400 Hz were included in the moderate pancreatic siderosis group, and those >400 Hz were included in the severe pancreatic siderosis group [14].

The degree of association between continuous and/or ordinal variables was calculated by Pearson correlation coefficient (r) and p<0.05 was considered significant.

## RESULTS

Eight patients with beta thalassemia major and 1 patient with NHL were included in this study (male/female: 5/4). The mean age of the patients was 17.4 years (range: 13-22 years). Thalassemia patients had a diagnosis of beta thalassemia major since infancy and were treated with repetitive red blood cell transfusion and iron chelation therapy. The patient with NHL had an 8-year disease history with two bone marrow transplantations and multiple red blood cell transfusion therapies. Three of the patients had a history of myocardial iron overload, and two of them had hypogonadotropic hypogonadism. The mean BMI of the patients was 19.1 kg/m2 (range: 17.6-20.7 kg/m2).

**Figure 1:**
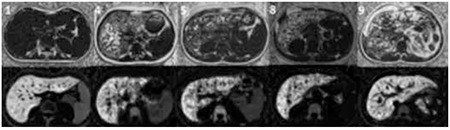
The magnetic resonance imaging-proton density fat fraction (top row) and R2* (bottom row) maps of the patients with severe hepatic iron overload. Except for Patient 1, all patients with severe hepatic iron overload demonstrated a patchy artifact in proton density fat fraction maps.

**Figure 2:**
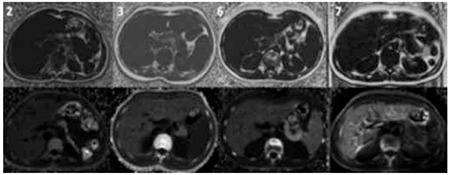
The magnetic resonance imaging-proton density fat fraction (top row) and R2* (bottom row) maps of the patients with milder hepatic iron overload. There is no artifact in patients with milder hepatic iron overload.

The mean ferritin level was 5933.1 ng/mL (range: 600-29950 ng/mL), mean liver R2* value was 424.8 Hz (range: 60.9-773.9 Hz), and mean liver MRI-PDFF was 22.4% (range: 1.5%-63.9%) in the study population. The results are summarized in Table 1. All of the patients had hepatic iron overload. Severe hepatic iron overload was recorded in 5/9 patients (56%). When we evaluated the PDFF maps of severe hepatic iron overload patients, we observed an extensive patchy artifact in the liver in the majority of them (4/5) (Figures 1 and 2). When we performed MRI-PDFF measurements even with these artifacts, we observed unexpectedly high MRI-PDFF values. The liver R2* values of these patients were higher (range: 671.1-773.9 Hz) when compared with the patient with severe hepatic iron overload without artifacts (528.70 Hz).

**Figure 3:**
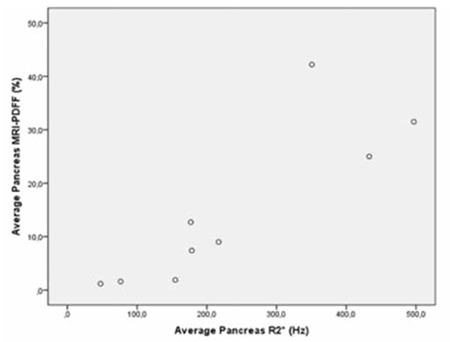
Scatterplot shows the correlation between pancreatic R2* and magnetic resonance imaging-proton density fat fraction (p=0.003, r=0.860).

Mean average pancreas R2* value was 236.8 Hz (47.5-496.9 Hz) and mean average pancreas MRI-PDFF value was 14.7% (1.2%-42.2%). All patients had pancreatic siderosis; 2 of them had mild siderosis, 5 of them had moderate siderosis, and 2 of them had severe siderosis (Table 2). There was a close correlation between average pancreas R2* and average pancreas MRI-PDFF (p=0.003, r=0.860) (Figure 3). We could not perform splenic measurements in 3 patients as they were splenectomized. Mean spleen R2* value was 142.1 Hz (29.9-224.2 Hz) and mean spleen MRI-PDFF was 2.1% (1.3%-3.1%). Mean vertebral bone marrow R2* value was 289 Hz (151.4-548.8 Hz) and mean vertebral bone marrow MRI-PDFF was 13.3% (0.1%-60%). Mean average renal cortex R2* value was 21.9 Hz (11.2-42.2 Hz) and mean average renal cortex MRI-PDFF was 0.8% (0%-1.5%).

**Figure 4:**
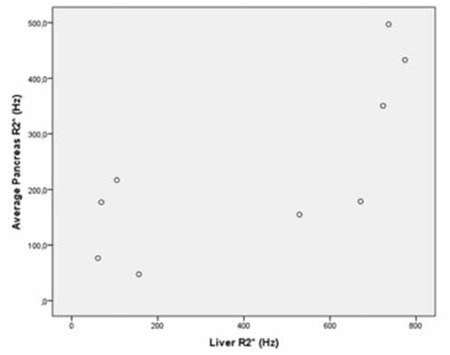
Scatterplot shows the correlation between liver R2* and average pancreas R2* (p=0.021, r=0,747).

**Figure 5:**
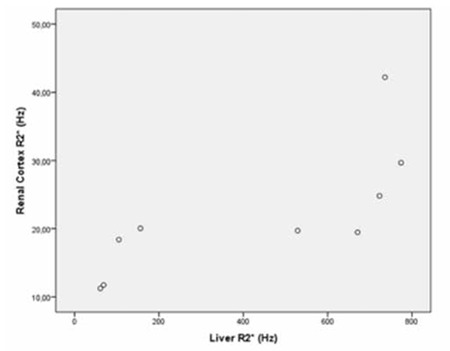
Scatterplot shows the correlation between liver R2* and renal cortex R2* (p=0.020, r=0.750).

**Figure 6:**
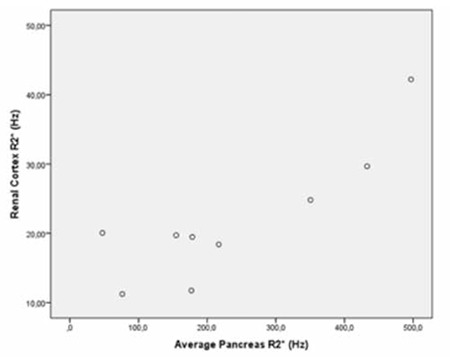
Scatterplot shows the correlation between average pancreas R2* and renal cortex R2* (p=0.003, r=0.858).

**Figure 7:**
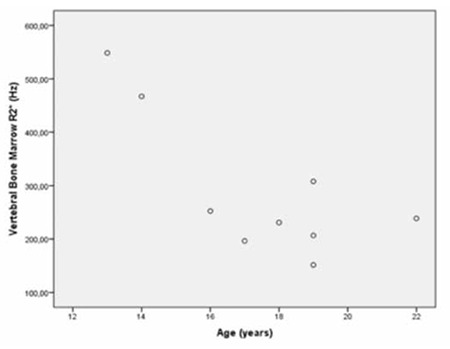
Scatterplot shows the correlation between vertebral bone marrow R2* and age (p=0.018, r=-0.759).

When we evaluated the correlations among patient age, serum ferritin level, and MRI findings, we observed a significant correlation between liver R2* and average pancreas R2* (p=0.021, r=0.747) (Figure 4). There was also a significant correlation among liver R2* and renal cortex R2* (p=0.020, r=0.750) (Figure 5) and average pancreas R2* and renal cortex R2* (p=0.003, r=0.858) (Figure 6). There was a significant negative correlation between vertebral bone marrow R2* and age (p=0.018, r=-0.759) (Figure 7). No other significant correlation was observed between patient age, serum ferritin level, and liver, pancreas, spleen, vertebral bone marrow, and renal cortex R2* and MRI-PDFF values.

## DISCUSSION

Noninvasive assessment of hepatic iron content in transfusion-related iron overload, which predominantly involves the pediatric population, is an important issue that was studied before by many investigators [6,7,8]. However, within the obesity epidemic, fat accumulation can coexist in the liver in such patients. The presence of both fat and iron in the liver has a confounding effect on the quantification of each one with MRI techniques [15]. In the present study we evaluated the feasibility of a recently described method, IDEAL-IQ, for liver, pancreas, spleen, vertebral bone marrow, and renal cortex iron and fat quantification in patients with transfusion-related iron overload.

Previously, Liau et al. evaluated the effect of changes in R2* caused by an intravenous infusion of super paramagnetic iron oxide contrast agent in quantification of liver fat fraction with IDEAL-IQ and observed that the IDEAL-IQ method of fat quantification is robust to changes in R2* [16]. However, the highest liver R2* value observed in this study was 212 Hz, which was distinctly lower than our patients with severe hepatic iron overload. Another study by Vasanawala et al. evaluated the clinical feasibility of weighted least squares T2* IDEAL, which is a similar technique to the one that we used, in transfusion-related iron overload and concluded that this technique is feasible in clinical applications [12]. In that study, investigators also observed significant hepatic steatosis in the liver in three patients that had milder hepatic iron overload compared with our study.

In our study, we observed artifacts and unexpectedly high MRI-PDFF values in PDFF maps of patients with a T2* value approximately under 1.6 ms with the MR-IDEAL technique. As discussed by Vasanawala et al. [12], estimation of a T2* value under the first echo time used in the technique is challenging. However, they did not mention that there is a problem in PDFF maps in such patients. In previous studies that evaluated the utility of this technique in quantification of hepatic steatosis in adults [17] and individuals predominantly consisting of pediatric patients [18] with biopsy-proven nonalcoholic fatty liver disease, the authors did not observe such high MRI-PDFF values, even in patients with severe steatosis, as we observed in the present study. Additionally, the patients in our study had no risk factors for hepatic steatosis or any hepatic steatosis, supporting additional imaging findings and confirming the inability of this technique in quantification of fat fraction in severe hepatic iron overload.

All of the patients in our study had pancreatic siderosis with variable degrees. Diabetes mellitus is one of the most common endocrine problems in thalassemia patients and pathogenesis depends on beta-cell dysfunction according to increased iron deposition [19]. It has been shown that early application of chelation therapy is protective for diabetes [20]. Pancreatic iron overload causes cell death and fatty transformation in pancreatic tissue [21]. An MRI technique that is feasible for both fat fraction quantification and R2* values would be valuable in assessing the pancreas in patients with transfusion-related iron overload. In our study, we could demonstrate both pancreatic siderosis and steatosis with MRI-PDFF. In addition, we observed a significant correlation between pancreatic siderosis and pancreatic steatosis, which was presumed but not demonstrated in previous studies.

Papakonstantinou et al. previously investigated the correlations between hepatic, splenic, pancreatic, vertebral bone marrow, and myocardial siderosis and did not find a correlation between pancreatic siderosis and hepatic and splenic siderosis [22]. Another study by Argyropoulou et al. did not find a correlation of T2 among the liver, bone marrow, pancreas, and pituitary gland [23]. However, in our study, we observed significant correlation between hepatic siderosis and pancreatic siderosis, hepatic siderosis, and renal cortical siderosis and pancreatic siderosis and renal cortical siderosis. We could not find a correlation between liver R2* and either spleen or bone marrow R2*. On the contrary, we observed a significant negative correlation between vertebral bone marrow R2* and age, which was not mentioned in previous studies.

One of the limitations of our study is the small sample size. However, there were patients with severe hepatic iron overload, which demonstrates the inability of the technique in fat quantification in short T2* values. Another limitation is the absence of histology assessment for accurate quantification of iron and fat content of the liver and other tissues. Furthermore, it is not feasible to evaluate iron and fat content of all organs with biopsy. On the other hand, we could easily demonstrate R2* values of different tissues just with an ROI placement with this technique, which is valuable in transfusion-related iron overload. In addition, we could demonstrate relationships between different tissues’ R2* values and the relationships between R2* values and fat fractions in tissues with a R2* value below approximately 671 Hz.

## CONCLUSION

High iron content of the liver, especially with a T2* value shorter than the first echo time can spoil the efficacy of PDFF calculation. Fat deposition in the pancreas is accompanied by pancreatic iron overload. There is a significant correlation between hepatic siderosis and pancreatic siderosis. Renal cortical and pancreatic siderosis are correlated, too.

## Figures and Tables

**Table 1:**
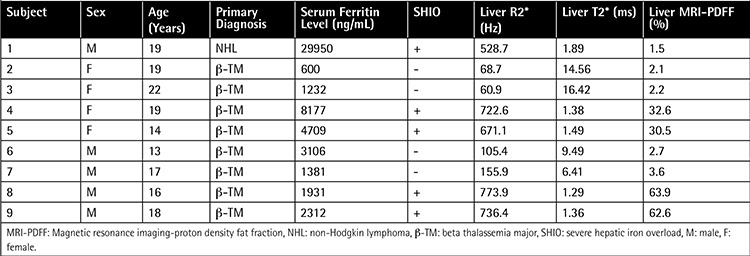
The characteristics and magnetic resonance imaging findings of patients with transfusion-related iron overload.

**Table 2:**
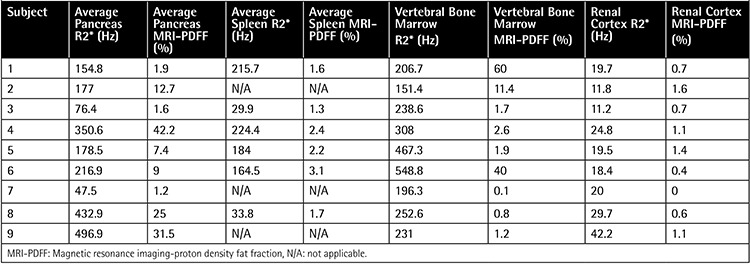
Pancreas, spleen, vertebral bone marrow, and renal cortical R2* and magnetic resonance imaging-proton density fat fraction of the patients.
